# Tannic acid-iron nanomaterial enhances rice growth and antioxidant defense under salt stress

**DOI:** 10.3389/fpls.2025.1565234

**Published:** 2025-07-11

**Authors:** Xiang Cheng

**Affiliations:** College of Bioscience and Biotechnology, Hunan Agricultural University, Changsha, China

**Keywords:** nanomaterial, rice, salt stress, reactive oxygen species, sustainable agriculture

## Abstract

Salinity stress severely impacts plant growth by reducing water uptake and biomass accumulation, while nanomaterial applications have emerged as effective solutions. This study introduces tannic acid-iron nanomaterial (TA-Fe Nanomaterial), a biocompatible nanomaterial synthesized via self-assembly, as a novel solution to mitigate salt stress. Characterized by lamellar morphology (200 nm average size) and robust thermal stability, TA-Fe Nanomaterial demonstrated potent reactive oxygen species (ROS) scavenging capabilities. Under 100 mM NaCl stress, applying 25 μ g/mL TA-Fe Nanomaterial enhanced rice seed germination, increasing root length by 85% compared to salt-stressed controls. In the hydroponic experiment, treated seedlings exhibited 70% and 87% increases in underground and aboveground lengths, alongside 133% higher fresh weight. Soil-cultivated rice showed 43–88% improvements in biomass and 67% greater shoot length. Furthermore, applying TA-Fe Nanomaterial can alleviate the aberrant ROS accumulation in leaves under the conditions of salinity stress. These findings suggest that TA-Fe Nanomaterial could be a promising tool for enhancing rice tolerance to salt stress, paving the way for future applications in sustainable agriculture.

## 1 Introduction

Rice (*Oryza sativa*) constitutes a vital cereal crop in China, contributing around 40% to the national grain production figures (Wang and Deng, 2018; Wan et al., 2022). In recent decades, as the phenomenon of soil salinization in China has become more and more serious, soil salinization has emerged as a predominant factor constraining crop growth. It is a severe problem facing the development of sustainable agriculture in China (Chen et al., 2021). According to the Food and Agriculture Organization (FAO), nearly 20% of the world’s irrigated land is affected by salinization, resulting in annual agricultural losses of billions of dollars and exacerbating global food security issues (Liu et al., 2022). Salt stress in saline-alkali soils reduces plants’ ability to absorb water from the soil. It leads to the accumulation of Na^+^ and Cl^-^ ions, causing metabolic disorders, oxidative damage, and nutritional imbalances in crops. In severe cases, it can even lead to plant death (Miller et al., 2010; Wungrampha et al., 2018). Traditional solutions to salinization primarily involve the use of fertilizers, freshwater irrigation, and conventional breeding methods. Although fertilizers can temporarily boost crop yields, they often result in severe environmental degradation (Kourgialas et al., 2017).

Moreover, agriculture dependent on freshwater irrigation can exacerbate soil salinization, leading to a sharp rise in dissolved salts over the past two decades due to the scarcity of high-quality irrigation water (Liu et al., 2020). Furthermore, traditional breeding methods have yielded limited advancements in creating crop varieties tolerant to salt (Ismail and Horie, 2017; Rawat et al., 2022). Therefore, there is an urgent need to engineer novel methodologies aimed at mitigating salinity stress in plants, improving crop yields, and advancing sustainable agricultural strategies.

Salt stress causes oxidative stress within plant systems. High salt concentrations produce large amounts of reactive oxygen species (ROS) within plant cells, and these reactive oxygen species, including hydrogen peroxide (H_2_O_2_) and superoxide anion (O_2_^-^). ROS are critical signaling molecules that activate plant defense responses to environmental stress (Mittler et al., 2022). Excessive accumulation of ROS can harm cell membranes, proteins, and DNA, leading to peroxidative degradation of lipids and oxidatively induced cellular damage in plant cells (Sahab et al., 2021; Zhang et al., 2022). In recent years, nanotechnology has been extensively utilized across various domains, such as biomedicine, mechanical equipment, and environmental protection. Nanoparticles have garnered considerable interest in agriculture owing to their enhanced uptake and penetration in plants, and their exceptional physicochemical properties arise from their nanoscale dimensions, distinctive architecture, and surface characteristics (Gupta et al., 2023). Nanoparticles, particularly metal oxide nanoparticles, have made significant progress in alleviating salt stress in plants. For example, titanium dioxide (TiO_2_) (Mustafa et al., 2022), zinc oxide (ZnO) (Ali et al., 2022), and iron oxide (Fe_2_O_3_) (Maswada et al., 2018) nanoparticles have demonstrated the ability to efficiently remove ROS generated within plant cells, reducing oxidative damage, enhancing salt tolerance, and protecting plant cells. In addition to promoting salt tolerance, these metal oxide nanoparticles can help maintain water and nutrient balance in plants (Zhao et al., 2022). Despite their success, some limitations remain, such as poor biocompatibility (Rossi et al., 2017; Song et al., 2023), high toxicity, and potential for inducing oxidative damage (Liu et al., 2021). Metal oxide nanoparticles, such as CeO_2_ (Rossi et al., 2016), Fe_2_O_3_ (Souza et al., 2019), and ZnO (Akanbi-Gada et al., 2019), used to enhance plant growth, may also pose risks of oxidative damage and growth inhibition. Hence, there exists a pressing requirement for the development of metal oxide nanomaterials that exhibit enhanced safety profiles and superior efficacy in ameliorating the detrimental impacts of salinity stress on plant life.

Tannic acid, also known as tannin, is a water-soluble polyphenolic compound with high biocompatibility. Extraction of tannic acid is feasible from various sources such as Vitis vinifera, Camellia sinensis, and fermented products of the grapevine. As a typical glucose-based compound, the polyphenolic hydroxyl groups in tannic acid endow it with unique chemical properties and physiological activities, including the ability to interact with proteins and polysaccharides to alter their performance (Ma et al., 2003). The catechol and galloyl groups in its structure give tannic acid antibacterial, antiviral, and antioxidant properties (Janicka et al., 2025). Moreover, it has excellent metal ion chelating and adsorption capabilities, along with superior biocompatibility, leading to its widespread application in food, healthcare, cosmetics, medicine, and leather industries (Kaczmarek-Szczepańska et al., 2023). In recent years, research teams globally have used tannic acid in nanoparticle production, developing various tannic acid-modified nanomaterials, such as polymethyl methacrylate nanoparticles (Marić et al., 2023), polyphonic acid nanoparticles (Sahiner and Sengel, 2016), targeted multifunctional tannic acid nanoparticles (Sahiner et al., 2016), iron tannic acid nanosheets (Aguilera et al., 2016), and modified tannic acid-iron (Pucci et al., 2022) nanomaterials. Among various types, nano-metals can be incorporated as nano-nutrient elements into rice seedlings to enhance plant growth effectively. Among them, FeS-NPs (iron sulfide nanoparticles) and MnS-NPs(manganese sulfide nanoparticles)have been demonstrated as effective nano-priming agents to promote the germination of naturally fungus-infected rice seeds. Metal-based nanomaterials have attracted extensive attention due to their unique catalytic properties, with iron-based nanomaterials exhibiting particularly outstanding performance (Revathy et al., 2023). Research has shown that iron-based nanomaterials not only enhance crop yields but also effectively control plant diseases, demonstrating extraordinary potential for applications in sustainable agricultural development (Hou et al., 2024). Furthermore, nanomaterials demonstrate remarkable advantages in the effective utilization of agricultural residues and the efficient production of biohydrogen (Cui et al., 2022). These innovative materials not only excel in drug delivery and environmental remediation but also exhibit outstanding biocompatibility and tunability.

Given the excellent properties and biological application potential of tannic acid, we designed a tannic acid-iron nanomaterial with high biocompatibility that is easy to synthesize. By neutralizing excess ROS generated under salt stress, TA-Fe Nanomaterial could protect rice cells from oxidative damage, maintain membrane integrity, and restore water-nutrient homeostasis, thereby alleviating the inhibitory effects of salinity on seed germination and seedling growth. In this study, we investigated the mitigating effect of TA-Fe nanomaterials on growth and development inhibition in rice under 100 mM NaCl stress(The NaCl solution concentration was set at 100 mM for the experiments, as this value corresponds to the mean concentration encountered in saline environments (Teige et al., 2004; Yang et al., 2019; Yang et al., 2021). Our goal was to investigate the potential application of TA-Fe Nanomaterial in enhancing plant salt tolerance, providing valuable insights for future research on using exogenous substances to mitigate salt stress in rice and offering new ideas for recreating saline-alkali soils in agriculture.

## 2 Materials and methods

### 2.1 Synthesis of TA - Fe nanomaterial

The TA-Fe nanomaterial was prepared using a self-assembly method: first, 100 μL of 24 mM tannic acid (TA; Macklin, Shanghai) and 100 μL of 24 mM ferric chloride hexahydrate (FeCl_3_·6H_2_O; Sinopharm) were dissolved in 50 mL of deionized water to obtain a uniformly dispersed suspension. The pH (PHS-25, Shanghai Yidian Scientific Instrument Co., Ltd.) was adjusted to 8 using NaOH (Sinopharm) under magnetic stirring (85-2, Changzhou Guoyu) for three hours. The suspension was centrifuged (8000 rpm, 5 min; TGL18W, Changsha Yingtai), washed thrice with deionized water, and then freeze-dried (FreeZone 6L, Labconco) to obtain the TA-Fe nanomaterial for subsequent experiments.

### 2.2 Antioxidant ability of TA-Fe nanomaterial

The antioxidant capacity of the TA-Fe nanomaterial (0-10 μg/mL) was tested using the 2,2-diphenyl-1-picrylhydrazyl method (DPPH; Adamas) to determine its ability to remove nitrogen-centered free radicals (Letelier et al., 2008). Different concentrations of TA-Fe nanomaterial (0-10 μg/mL) were mixed with 0.05 mg/mL DPPH in ethanol. After 30 min of dark incubation, absorbance was measured at 517 nm (Epoch microplate reader, BioTek).

The ability to scavenge superoxide anions was measured using the nitro blue tetrazolium method (NBT; Adamas) (Van Noorden and Butcher, 1989). Riboflavin (200 μM, 20 μL; Macklin, Shanghai), methionine (125 mM, 20 μL; Adamas), nitro blue tetrazolium (750 μM, 20 μL), and different concentrations of TA-Fe nanomaterial (0-10 μg/mL) were mixed in PB (pH 7.4, 10 mM) and illuminated (12,000 Lux) for 1 hour. Absorbance at 560 nm was recorded.

The ability to scavenge hydroxyl radicals was assessed using the Fenton reaction (Sui et al., 2024). Ferrous sulfate (4 μM, 10 μL), 10 μL of 20 mM salicylic acid (SA; Sinophar), 10 μL of 20 mM hydrogen peroxide (H_2_O_2_; Sinopharm), and different concentrations of TA-Fe nanomaterial (0-10 μg/mL) were reacted in ultrapure water (Ultrapure water system, VIP12-UV, Hunan Colton Waterworks Co., Ltd.) for 20 minutes. Absorbance was quantified at a wavelength of 510 nm.

The capacity to scavenge hydrogen peroxide was determined by employing UV spectrophotometry (UV-3600 Plus, Shimadzu). H_2_O_2_ (200 mM, 20 μL), Phosphate Buffered Saline (pH 7.4, 10 mM; PBS; Bioss, Beijing Bioss Biotech Co., Ltd.), and different concentrations of TA-Fe nanomaterial (0-10 μg/mL) were reacted in ultrapure water under dark conditions for a period of 6 hours, and absorbance was measured at 240 nm.

### 2.3 Rice seed soaking experiment with TA-Fe nanomaterial

Healthy, intact “Xiu Zhan 15” rice seeds (Zhongken Jinxiu Huanong Wuhan Technology Co., Ltd.) were soaked in 20 mL of tap water, NaCl solution (20 mL, 100 mM; Sinopharm), three different concentrations of NaCl + TA-Fe nanomaterial solution (20 mL, NaCl: 100 mM; TA-Fe nanomaterial: 12.5, 25, 50 μg/mL) at room temperature. After three days, the germination of rice seeds in each group was observed, and the root length of each group was measured. See Section 2.6 for details on the experimental design.

### 2.4 Hydroponic experiment with TA-Fe nanomaterial

Healthy and intact “Xiu Zhan 15” rice seeds were selected and placed into five separate hydroponic containers (1 L) for cultivation. All groups were initially cultivated with tap water for the first three days. From the fourth day onward, the treatment solutions were replaced as follows: the blank control group (Water) continued with tap water, the salt stress group (NaCl) was replaced with 100 mM NaCl solution, and three treatment groups were replaced with solutions containing 100 mM NaCl and gradient concentrations of TA-Fe nanomaterial (12.5, 25, and 50 μg/mL). All treatment solutions were adjusted to a final volume of 1 L. The hydroponic boxes were maintained at room temperature, with daily replenishment of evaporated water to maintain a constant liquid level. The solutions were completely replaced with fresh treatment solutions every 48 hours, and growth progress was observed and photographed. After ten days of cultivation, six rice seedlings were randomly selected from each group to determine the length and fresh weight of the underground and aboveground parts. The seedlings were then desiccated in an electric blast drying oven (BGZ-240, Shanghai Boxun Medical Biological Instrument Co., Ltd.) maintained at a temperature of 80°C for 36 hours, after which the dry weight was determined. See Section 2.6 for details on the experimental design.

### 2.5 Soil cultivation experiment with TA-Fe nanomaterial

Germinated seeds of the “Xiu Zhan 15” variety, with similar health and growth status, were transplanted into plastic pots containing 500 g of soil, which were divided into five groups(Water, NaCl, NaCl + TA-Fe nanomaterial: 12.5, 25, 50 μg/mL). On the fourth day, watering began, with 50 mL of the respective solutions provided daily. Seedling growth was monitored and photographed regularly. On the 13th day, four rice seedlings from each group were randomly selected to determine the length and fresh weight of both underground and aboveground parts. Subsequently, the seedlings from each group underwent desiccation in an oven maintained at 80°C for 36 hours, and the dry weight and dry-to-fresh weight ratios were calculated.

The assessment of cell membrane integrity and oxidative stress levels in rice seedlings under salt stress was conducted using Evans Blue (EB; Adamas) with 3,3’-diaminobenzidine (DAB; Macklin) staining.

In the context of EB staining, the integrity of cell membranes was detected by applying a non-permeable dye, EB (0.25%), which specifically stains dead cells with impaired membrane structure (dark blue) (Baker and Mock, 1994). Intact cells were found to be unable to take up the dye due to the membrane barrier (light blue or no staining). This method intuitively reflects the degree of damage to the cell membrane by salt stress and the protective effect of TA-Fe nanomaterials.

DAB staining: The presence of H_2_O_2_ in tissues was determined through the use of a DAB (1 mg/mL, pH 3.8) staining procedure. The DAB staining method revealed a positive correlation between the intensity of the staining and the amount of H_2_O_2_ present. This reaction occurred due to the reaction of DAB with peroxidase in the presence of H_2_O_2_, resulting in the formation of a brown precipitate (Forney et al., 1982). This method was employed to quantify the oxidative stress induced by salt stress and the antioxidant capacity of TA-Fe nanomaterials.

For staining experiments, the remaining seedlings’ aboveground parts were immersed in DAB (1 mg/mL, pH adjusted to 3.8 with hydrochloric acid) or EB (0.25%) for 24 hours in the dark. After staining, the samples were rinsed with deionized water and boiled in anhydrous ethanol (Hengxing Reagent, Tianjin Hengxing Chemical Reagent Manufacturing Co., Ltd.) until most of the green color faded from the leaves, and then the staining solutions were separately extracted from leaves of rice seedlings across all treatment groups, maintaining consistent sampling positions between biological replicates. For experimental design details, see Section 2.6.

### 2.6 Experimental design

All experiments were conducted using a completely randomized design (CRD) to ensure unbiased allocation of treatments. The experimental setup is detailed as follows:

#### 2.6.1 Setting treatment groups

The soaking, hydroponics and soil culture experiments were divided into five groups; each group treated twenty healthy and intact Xiuzhan 15 rice seeds as follows:

1. Control group (Water): No salt stress or nanomaterial application.
2. Salt Stress group (NaCl): 100 mM NaCl to simulate saline conditions.
3. Salt stress + TA-Fe nanomaterials low concentration group: 100 mM NaCl + 12.5 μg/mL TA-Fe nanomaterials.
4. Salt stress + TA-Fe nanomaterials medium concentration group: 100 mM NaCl + 25 μg/mL TA-Fe nanomaterials.
5. Salt stress + TA-Fe nanomaterials high concentration group: 100 mM NaCl + 50 μg/mL TA-Fe nanomaterials.

#### 2.6.2 Replication and randomization

Seed Soaking Experiment (Section 2.3):

Sample size and treatment: twenty seeds in each group were soaked in 20 mL of the corresponding treatment solution (tap water, 100 mM NaCl solution, or a mixture containing graded concentrations of TA-Fe nanomaterials).

Repeat setting: each group was repeated three times (total of sixty seeds/treatment).

Observation index: record the germination rate after 3 days of soaking and measure the root length of all seeds.

Hydroponic Experiment (Section 2.4):

Sample size and treatments: twenty seedlings in each group were cultured in a 1 L hydroponic system, using tap water for the first three days and replacing it with the corresponding treatment solution on the fourth day.

Random sampling and measurements: after ten days of incubation, six plants were randomly selected from twenty plants in each group, and their underground and aboveground length and fresh weight were measured, dried, and the dry weight was determined and the dry-to-fresh ratio was calculated.

Replications: Each group was set up with three independent hydroponic systems (technical replications).

Soil Cultivation Experiment (Section 2.5):

Sample size and treatments: twenty seedlings in each group were transplanted into plastic pots containing 500 grams of soil and watered daily with 50 mL of the corresponding treatment solution from day four onwards.

Random sampling and measurements: after thirteen days of incubation, four plants from twenty plants in each group were randomly selected to measure the length and fresh weight of the underground and above ground, dry weight was determined after drying and dry-fresh ratio was calculated.

Replication setting: each group was replicated four times (biological replication).

#### 2.6.3 Quantification methods

##### 2.6.3.1 EB staining (cell viability)

Control subjects: Heat-killed negative controls were established by boiling water bath treatment (100°C, 15–20 min) to induce complete cellular mortality.

Absorbance measurement: 610 nm

Dual quantification system:

(a) Morphometric analysis: The length of the leaves non-staining fragment (L_n_) was calculated against the total length of the seedlings (L_t_). The ratio of the two was taken as the relative cell viability (V): V = L_n_/L_t_ × 100%

(b) Spectrophotometric calculation: The formula V = [1 - A/A_d_ × 100% was established to calculate the relative cell viability (V), where A is the absorbance of the treatment groups, and A_d_ is the absorbance of the leaves boiled to death.

##### 2.6.3.2 DAB staining (H_2_O_2_ detection)

Control subjects: NaCl-treated seedlings

Absorbance measurement: 450 nm

Dual quantification system:

(a) Morphometric analysis: The length of the leaves staining fragment (L) was calculated against the total length of the seedlings (L_t_). The ratio of the two was taken as the relative hydrogen peroxide content (R_c_): R_c_ = L/L_t_ × 100%

(b) Spectrophotometric calculation: The formula R_c_ = A/AN×100% was established to calculate the relative hydrogen peroxide content (R_c_), where A is the absorbance of the treatment groups, and AN is the absorbance of the NaCl-treated seedlings.

#### 2.6.4 Statistical analysis

Data are presented as mean ± standard deviation (SD) from at least three independent biological replicates. Statistical significance was determined by one-way ANOVA followed by Tukey’s *post hoc* test (α=0.05). Error bars in all figures represent SD.

#### 2.6.5 Dose-response assessment

In the seed soaking experiment, additional concentrations of TA-Fe Nanomaterial (12.5 and 50 μg/mL) were tested alongside the optimal 25 μg/mL dose to evaluate dose-dependent effects.

## 3 Results

### 3.1Characterization of TA-Fe nanomaterial

**Figure 1:**
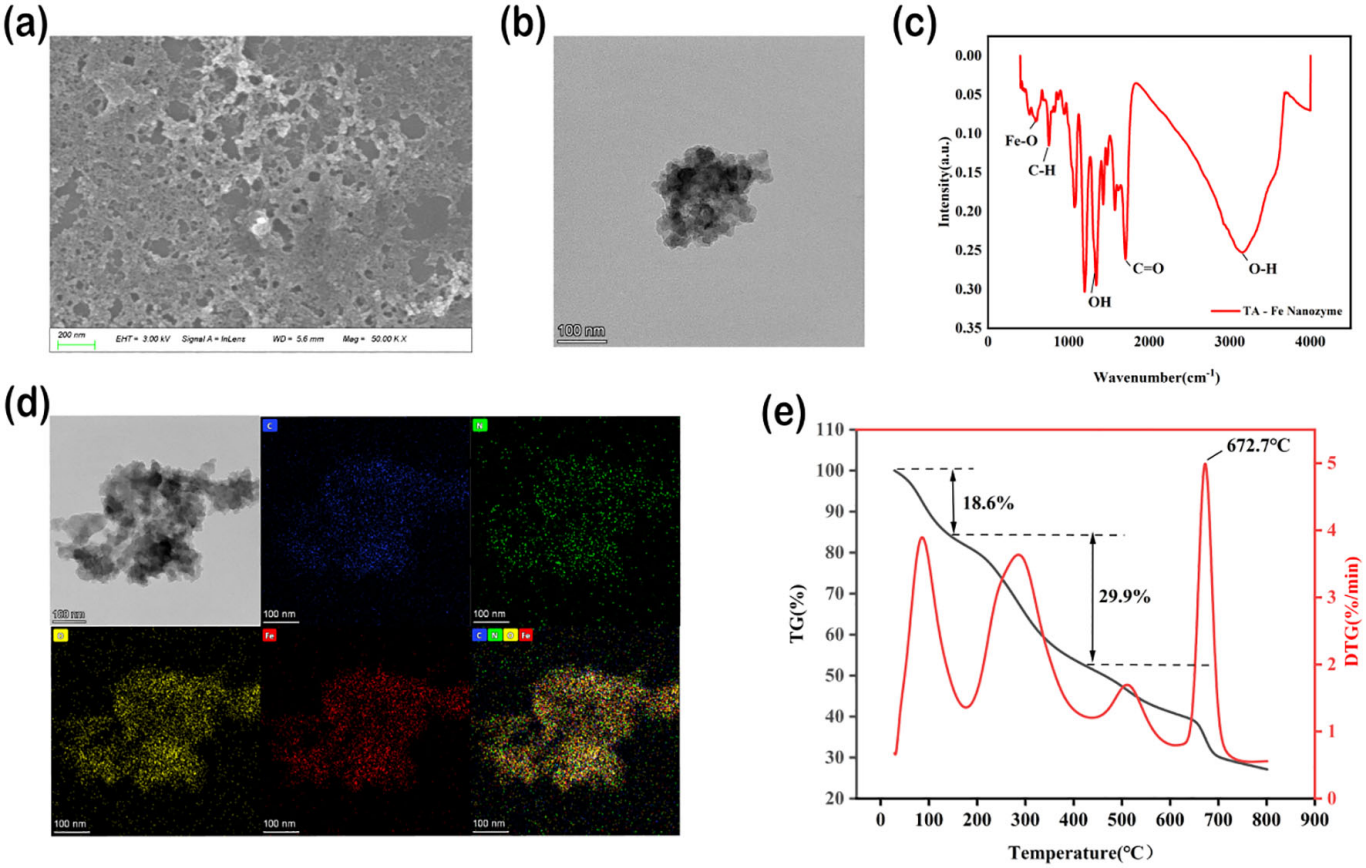
Characterization of physicochemical properties of TA-Fe nanomaterial. **(a)** SEM image showing lamellar morphology. **(b)** TEM image. **(c)** FTIR spectrum confirms Fe-O bond formation (594 cm-¹). **(d)** TEM image of TA-Fe Nanomaterial and elemental mappings of C, N, O, and Fe, respectively. **(e)** TGA analysis showed the thermal stability of the material (onset of weight loss 672.7°C).

TA - Fe nanomaterials were synthesized by complexation. Transmission electron microscopy (FEI Tecnai F20 TEM, Thermo Fisher Scientific, USA.) and scanning electron microscopy (ZEISS Sigma 300 field-emission SEM, Carl Zeiss AG, Germany.) analyses showed that TA-Fe nanomaterials displayed a lamellar morphology with a distinct distribution with an average size of 200 nm and 100 nm (**Figures 1a, b**). Elemental mapping validation of the constituent elements indicated the presence of C, N, O, and Fe on the substratum of TA - Fe nanomaterials (**Figure 1d**). Meanwhile, Fourier transform infrared (FTIR) spectra showed that the peak located at 3149 cm^-1^ corresponded to the characteristic peak of O-H in TA. The characteristic peaks of C-H and C-O appeared at 755 cm^-1^ and 1705 cm^-1^, respectively. Notably, the appearance of the characteristic peak of Fe-O (594 cm^-1^) confirms the successful reaction of Fe-O with TA (**Figure 1c**). TGA was used to study the thermal behavior of TA-Fe nanomaterials (**Figure 1e**). TGA is used to study the thermal behavior of Cs−Se nanomaterials and demonstrates how chemical transformations affect it.

TA-Fe nanomaterials lost weight in three stages (**Figure 1e**). The mass of TA-Fe nanomaterials changed markedly only when the temperature reached 672.7°C, suggesting that they exhibit superior thermal stability.

### 3.2 Antioxidant capacity of TA-Fe nanomaterial

**Figure 2:**
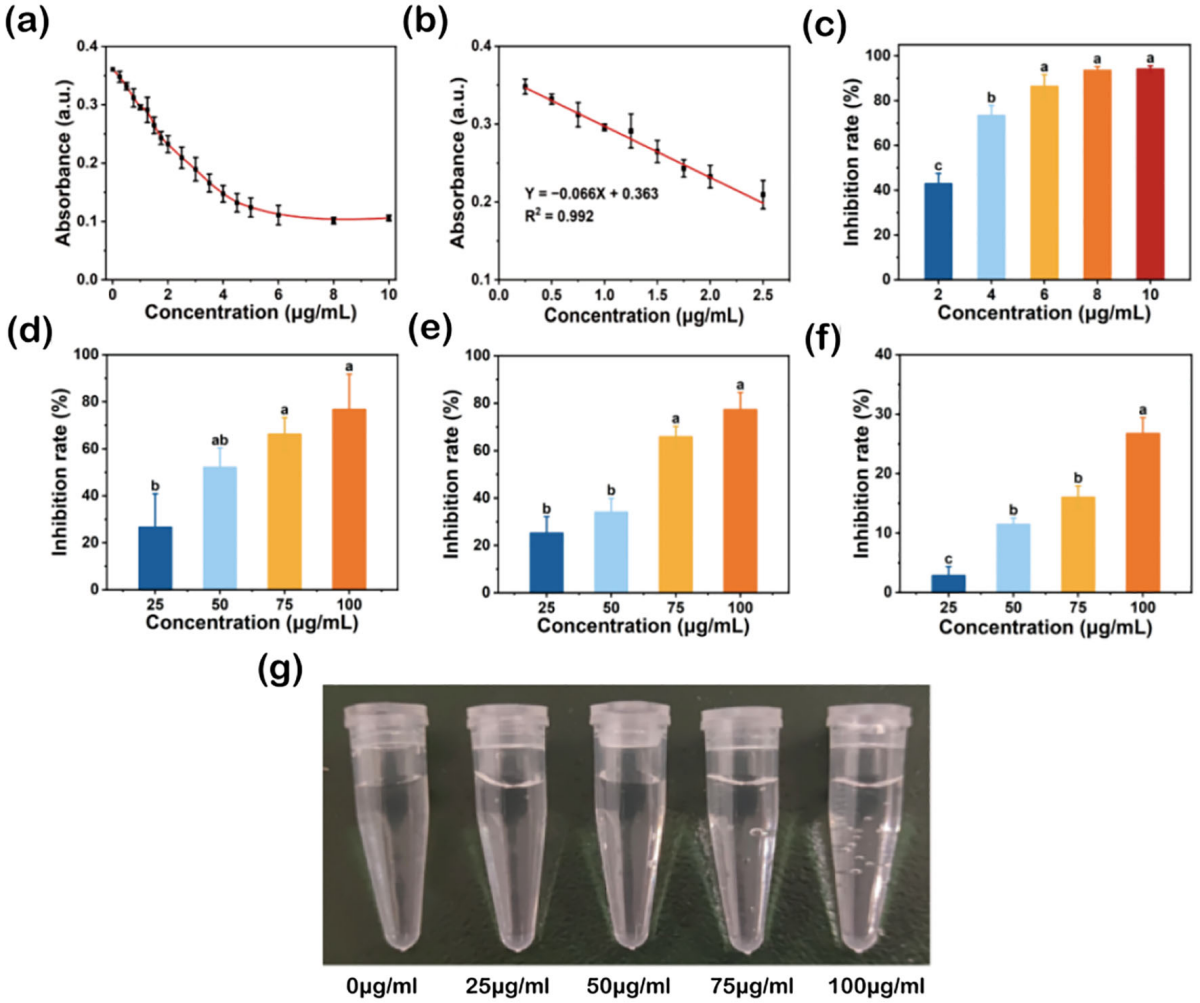
Broad-spectrum free radical scavenging ability of TA-Fe nanomaterial. **(a)** Effect of different concentrations of TA - Fe Nanomaterial on the absorbance of DPPH at 517 nm and **(b)** linear fit image with low concentration. **(c)** Inhibition rates and multiple comparisons (α = 0.05) of DPPH (86.36% inhibition rate at 100 μg/mL), **(d)** ·O_2_^-^ (76.68% inhibition rate at 100 μg/mL), **(e)**·OH (77.21% inhibition rate at 100 μg/mL) and **(f)** H_2_O_2_ (26.72% inhibition rate at 100 μg/mL). **(g)** Effect of TA - Fe Nanomaterial concentration (from left to right, 0, 25, 50, 75 and 100 μg/mL) on the decomposition of H_2_O_2_ to produce oxygen bubbles. Error bars denote SD (n=3). Identical lowercase letters (e.g., a, a) indicate no significant difference (p > 0.05). Different lowercase letters (e.g., a, b) indicate statistically significant differences (p < 0.05).

The high antioxidant activity of TA-Fe nanomaterial is reflected in its broad-spectrum ability to scavenge ROS and RNS and stabilize free radicals. The DPPH radical is a stable nitrogen-centered free radical with an absorption peak at 517 nm. When a radical scavenger is present, the absorbance of DPPH decreases, allowing the antioxidant capacity of the scavenger to be quantitatively analyzed (Markus et al., 2025). The effect of different concentrations of TA-Fe nanomaterial on DPPH radical scavenging is shown in **Figures 2a–c**. Generally, as the concentration of TA-Fe nanomaterial increased, the absorbance at 517 nm decreased, with the reduction in absorbance diminishing significantly at concentrations above 6 μg/m L. Moreover, at concentrations below 2.5 μg/mL, the absorbance had a linear relationship with the concentration. At concentrations of 2, 4, 6, 8, and 10 μg/mL, the inhibition rates of DPPH were 42.92%, 73.35%, 86.36%, 93.48%, and 94.04%, respectively. Significant differences were observed between the inhibition rates at 2, 4, and 6 μg/mL. Overall, the TA-Fe nanomaterial demonstrated strong nitrogen-centered free radical scavenging ability at concentrations below 6 μg/mL.

Superoxide anions (·O_2_−) are reactive oxygen species (ROS) that can be catalyzed by superoxide dismutase (SOD) to form oxygen and hydrogen peroxide in biological systems. Under environmental stress, ROS levels surge, disrupting the balance between oxidation and antioxidation, ultimately damaging cells. Flavin and methionine can generate superoxide anions under light, which react with NBT to produce a blue-colored substance that strongly absorbs at 560 nm (Dondurmacıoğlu et al., 2017). By introducing superoxide anion scavengers, the production of this colored substance can be reduced, providing a quantitative assessment of antioxidant performance. The effect of different concentrations of TA-Fe nanomaterial on superoxide anion scavenging is shown in **Figure 2d**. With increasing TA-Fe nanomaterial concentration, the scavenging effect of superoxide anions was enhanced. At concentrations of 25, 50, 75, and 100 μg/mL, the inhibition rates of superoxide anions were 26.57%, 52.10%, 66.19%, and 76.68%, respectively. Significant differences were o noted at concentrations of 75 and 100 μg/mL compared to 25 μg/m L. This experiment confirmed that the TA-Fe nanomaterial has a strong capacity to scavenge superoxide anions.

Hydroxyl radicals (·OH) are highly reactive oxygen species. The principle of the Fenton reaction is based on the catalytic decomposition of hydrogen peroxide by ferrous ions to produce hydroxyl radicals, which can oxidize organic pollutants in wastewater. SA, an organic acid, can be oxidized by hydroxyl radicals to form a substance that strongly absorbs at 510 nm. By adding hydroxyl radical scavengers, the production of this colored substance can be reduced, allowing the quantitative assessment of antioxidant capacity (Rutely et al., 2018). The effect of different concentrations of TA-Fe nanomaterial on hydroxyl radical scavenging is shown in **Figure 2e**. Overall, increasing TA-Fe nanomaterial concentrations enhanced the scavenging of hydroxyl radicals. At concentrations of 25, 50, 75, and 100 μg/mL, the inhibition rates of hydroxyl radicals were 25.15%, 34.00%, 65.78%, and 77.21%, respectively. Significant differences were observed between the concentrations of 75 and 100 μg/mL compared to lower concentrations, confirming that the TA-Fe nanomaterial is highly effective at scavenging hydroxyl radicals.

Hydrogen peroxide (H_2_O_2_) is another ROS commonly found in biological systems. It can easily penetrate cell membranes and has strong cytotoxicity. In biological systems, catalase (CAT) breaks down H_2_O_2_ into oxygen and water. H_2_O_2_ absorbs at 240 nm, and its concentration can be measured using UV spectrophotometry (Hamza and Hadwan, 2020). The scavenging effect of TA-Fe nanomaterial on H_2_O_2_ is shown in **Figure 2f**. With the increment in TA-Fe nanomaterial concentration, the scavenging efficiency of H_2_O_2_ also improved. At concentrations of 25, 50, 75, and 100 μg/mL, the inhibition rates of H_2_O_2_ were 2.85%, 11.42%, 15.99%, and 26.72%, respectively. Additionally, the formation of oxygen bubbles due to the decomposition of H_2_O_2_ was observed in EP tubes as the elevation of TA-Fe nanomaterial concentration (**Figure 2g**). Overall, the results demonstrate that the TA-Fe nanomaterial effectively catalyzes the decomposition of H_2_O_2_, yielding water and oxygen as products.

In summary, the TA-Fe nanomaterial exhibited strong antioxidant properties *in vitro*, demonstrating the ability to effectively scavenge nitrogen-centered free radicals, superoxide anions, hydroxyl radicals, and hydrogen peroxide.

### 3.3 Analysis of rice seed soaking results with TA-Fe nanomaterial

**Figure 3:**
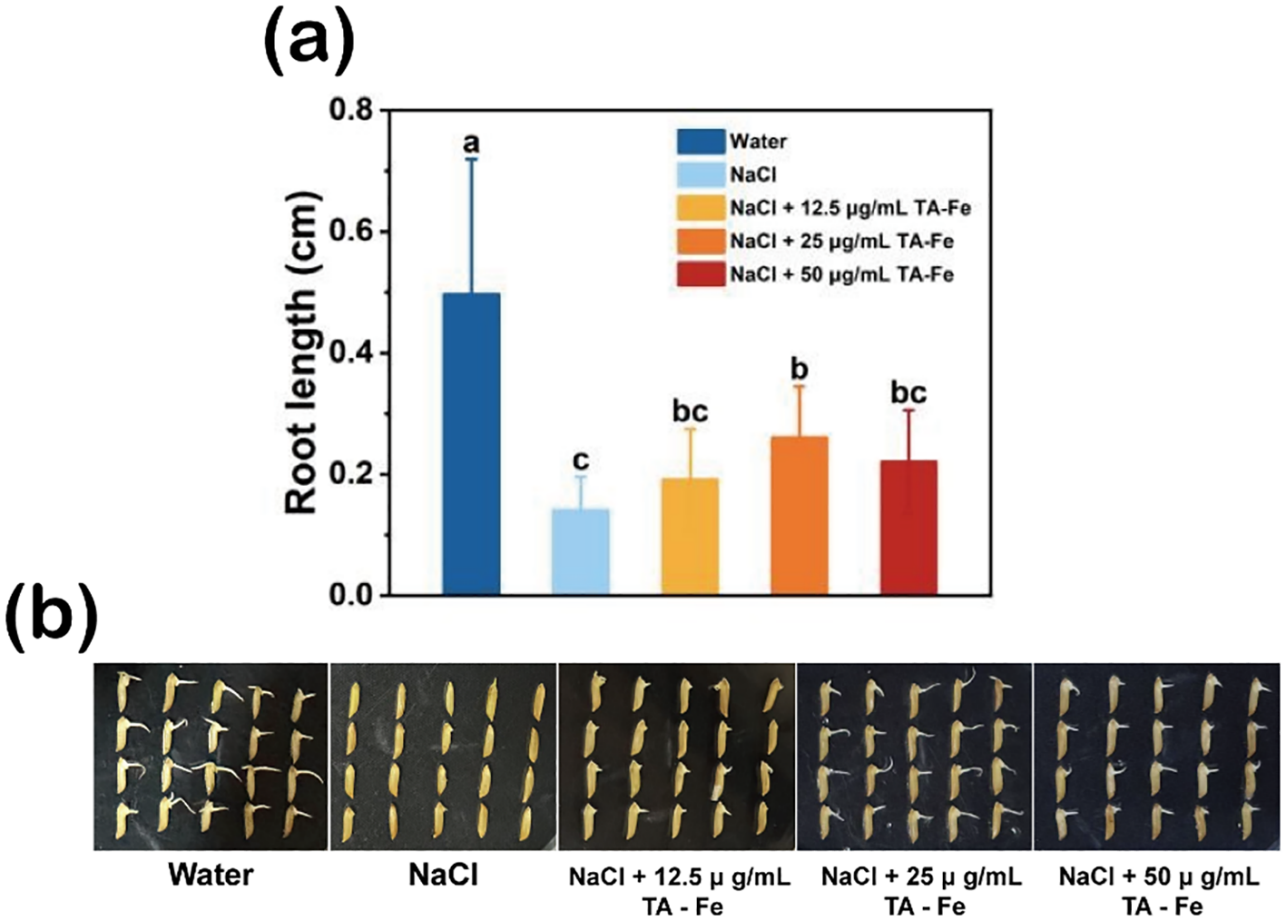
TA-Fe nanomaterial mitigates the inhibition of salt stress on seed germination and root length in rice. **(a)** Effect of different concentrations of TA-Fe nanomaterial on root length of rice under salt stress (α = 0.05). **(b)** Photographs of germinated rice seeds (from left to right, Water, NaCl, NaCl + 12.5 μ g/mL TA - Fe Nanomaterial, NaCl + 25 μ g/mL TA - Fe Nanomaterial, NaCl + 50 μ g/mL TA - Fe Nanomaterial). Error bars denote SD (n=20). Identical lowercase letters (e.g., a, a) indicate no significant difference (p > 0.05). Different lowercase letters (e.g., a, b) indicate statistically significant differences (p < 0.05).

The rice variety “Xiu Zhan 15” was used to study the effect of TA-Fe nanomaterial on rice seed germination under salt-stress conditions. The results are shown in **Figure 3**. After soaking for three days, the average root length of the control group (water group) was 0.49 cm, significantly longer than that of the salt-stress group (0.14 cm), the salt stress + 12.5 μg/mL TA-Fe nanomaterial group (0.19 cm), the salt stress + 25 μg/mL TA-Fe nanomaterial group (0.26 cm), and the salt stress + 50 μ g/mL TA-Fe nanomaterial group (0.22 cm).

Among these, the average root length of the salt stress + 25 μg/mL TA-Fe nanomaterial group was approximately 85% longer than that of the salt stress group. Although the other TA-Fe nanomaterial groups had longer average root lengths than the salt stress group, the differences were insignificant. Furthermore, the average root length in the salt stress + 50 μg/mL TA-Fe nanomaterial group was shorter than that of the 25 μg/mL group, suggesting a possible toxic effect due to an excess of TA-Fe nanomaterial. It indicates that the treatment with 25 μg/mL TA-Fe nanomaterial can substantially ameliorate the inhibitory effect of salinity stress on rice seed root development. Overall, salinity stress exerted a profound inhibitory effect on the germination of rice seeds and the growth of their roots. At the same time, the application of 25 μg/mL TA-Fe nanomaterial mitigated this inhibition.

### 3.4 Analysis of hydroponic rice results with TA-Fe nanomaterial

**Figure 4:**
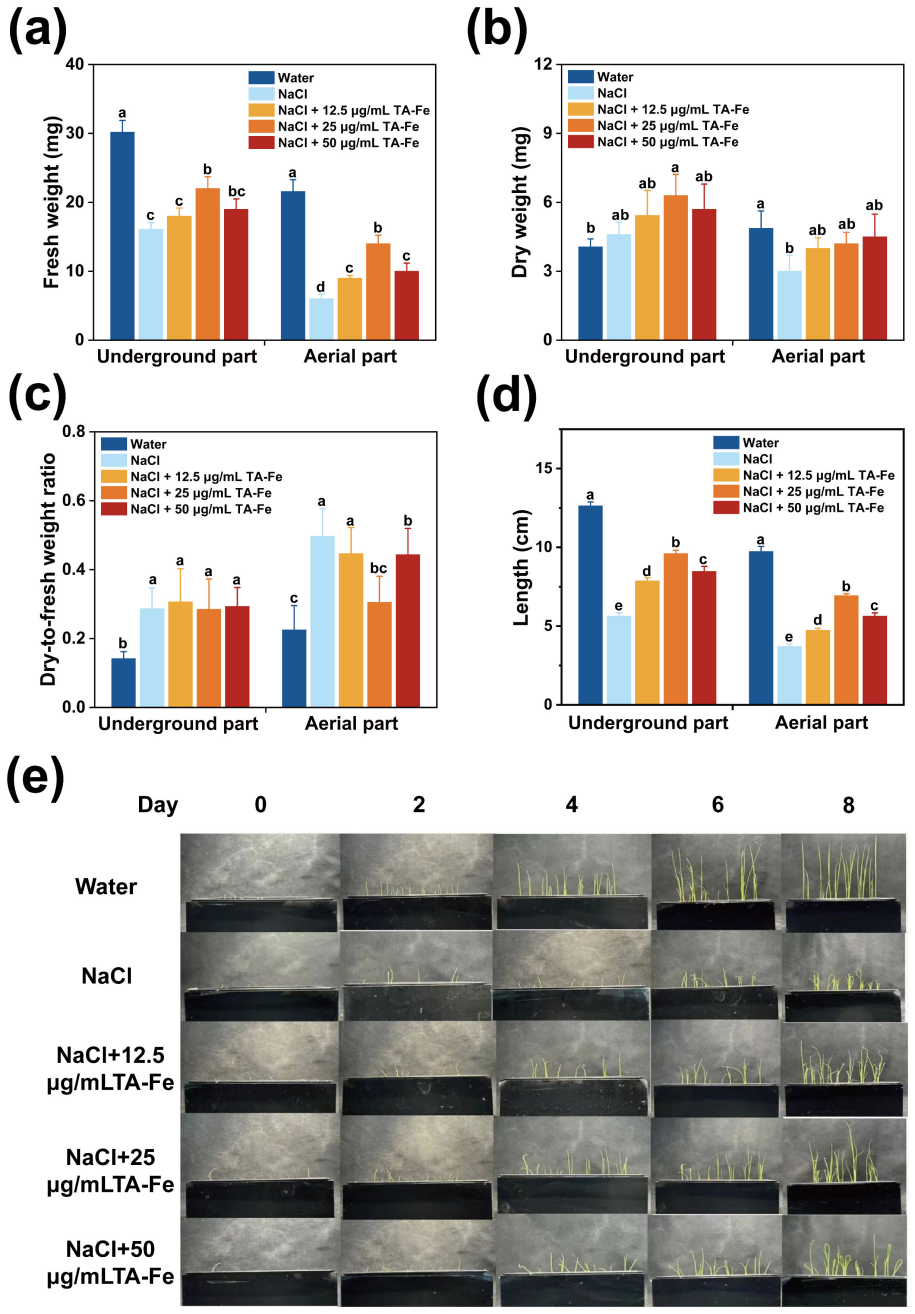
Regulation of growth parameters of hydroponic rice under salt stress by TA-Fe nanomaterial. **(a)** Fresh weight, **(b)** dry weight, **(c)** dry-to-fresh weight ratio and **(d)** length of the underground and aerial parts of hydroponic rice and multiple comparisons (α = 0.05) in the same part. **(e)** Promotional effect of TA-Fe nanomaterial on the growth of rice seedlings under salt stress (from top to bottom, Water, NaCl, NaCl + 12.5 μg/mL TA - Fe Nanomaterial, NaCl + 25 μg/mL TA - Fe Nanomaterial, NaCl + 50 μg/mL TA - Fe Nanomaterial, n=20). Error bars denote SD (n=6). Identical lowercase letters (e.g., a, a) indicate no significant difference (p > 0.05). Different lowercase letters (e.g., a, b) indicate statistically significant differences (p < 0.05).

In this experiment, the “Xiu Zhan 15” rice variety was selected to investigate the effects of TA-Fe nanomaterial on rice under salt stress conditions in a hydroponic culture. The results are presented in **Figure 4**.

After ten days of cultivation in the hydroponic system, the average lengths of the underground and aboveground parts in the water group were 12.63 cm and 9.73 cm, respectively, while those in the NaCl group decreased to 5.63 cm (55% decrease) and 3.7 cm (62% decrease), respectively (**Figure 4d**). These experimental results indicated that salt stress caused by 100 mM NaCl could significantly inhibit rice growth.

Adding 12.5 μg/mL of TA-Fe Nanomaterial restored the underground length to 8.53 cm (52% higher than that of the NaCl group), and the aboveground fresh weight reached 9 mg (50% higher than that of NaCl), but the underground fresh weight of 18 mg was still significantly lower than that of the water group, indicating that the low concentration only partially alleviated the stress. The best results were obtained in the 25 μg/mL TA-Fe nanomaterial group, where the underground length increased to 9.6 cm (70% higher than that of NaCl), which was close to 76% of that of the water group. The aboveground fresh weight increased to 14 mg (133% higher than that of the NaCl group), indicating that the medium concentration was effective in neutralizing salt stress by promoting root elongation and biomass accumulation. However, the below-ground length (8.47 cm) and above-ground fresh weight (10 mg) were less increased in the 50 μg/mL TA-Fe nanomaterial addition group than in the 25 μg/mL group, possibly due to the high concentration of nanomaterial aggregation or slight toxicity limiting its efficacy (**Figures 4a, d**).

Furthermore, the underground part in the water group had a lower dry weight and dry-to-fresh weight ratio, indicating a higher water content, better vitality of the seedlings, and effective utilization of dry matter from the endosperm. There was no marked difference in these parameters between the Salt Stress and Salt Stress + TA-Fe nanomaterial groups, which suggests that the salt stress impacted both.

The dry weight data for the aboveground parts revealed that the Water group accumulated the most dry matter, while the Salt Stress group accumulated the least (**Figure 4b**). Significant differences were found in pairwise comparisons between the three groups, highlighting the suppressive impact of saline stress on aboveground dry matter accumulation. However, the application of 25 μg/mL

TA-Fe nanomaterial alleviated this inhibition, as indicated by the increase in aboveground dry weight. The dry-to-fresh weight ratio for the aboveground parts indicated that the Water group had higher water content and vitality (**Figure 4c**). In contrast, salt stress reduced water content and inhibited seedling growth, while the addition of 25 μg/mL TA-Fe nanomaterial increased water content, alleviating this inhibitory effect.

In conclusion, the hydroponic experimental data indicate that the supplementation of 25 μg/mL TA-Fe nanomaterial significantly mitigates the detrimental effects of salt stress on various aspects of rice growth in hydroponic culture, improving plant height, fresh weight, and aboveground dry weight.

### 3.5 Analysis of rice soil cultivation results with TA-Fe nanomaterial

**Figure 5:**
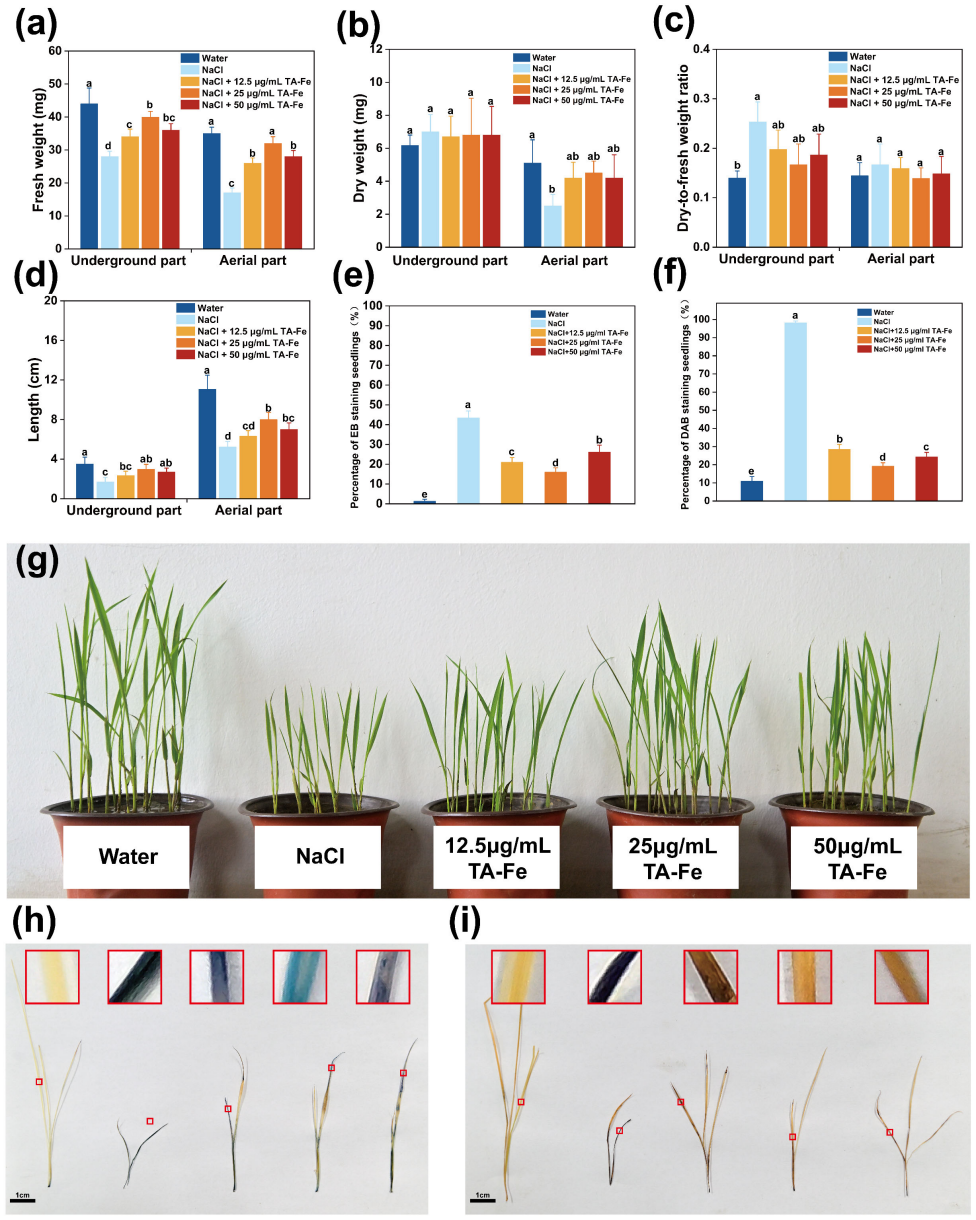
TA-Fe nanomaterial enhances salt tolerance in rice by reducing oxidative damage. **(a)** Fresh weight, **(b)** dry weight, **(c)** dry-to-fresh weight ratio and **(d)** length of the underground and aerial parts of soil cultivated rice and multiple comparisons (α = 0.05) in the same part. **(e)** Percentage of EB staining seedlings (%). **(f)** Percentage of DAB staining seedlings (%). **(g)** Photographs during soil cultivation (from left to right, Water, NaCl, NaCl + 12.5 μg/mL TA - Fe Nanomaterial, NaCl + 25 μg/mL TA - Fe Nanomaterial, NaCl + 50 μg/mL TA - Fe Nanomaterial, n=20). **(h)** EB staining shows membrane damage in the salt stress group (dark blue) versus membrane integrity in the TA-Fe group (light blue). **(i)** DAB staining showed that TA-Fe treatment significantly reduced H_2_O_2_ accumulation (light yellow vs. dark brown in the salt stress group). Error bars denote SD (n=4). Identical lowercase letters (e.g., a, a) indicate no significant difference (p > 0.05). Different lowercase letters (e.g., a, b) indicate statistically significant differences (p < 0.05).

In this experiment, the rice variety “Xiuzhan 15” was selected to study the effects of TA-Fe nanomaterial under salt stress conditions. The results are presented in **Figure 5**. After nine days of various treatments, the underground fresh weight (28 mg) and aboveground fresh weight (17 mg) of the NaCl group were only 64% and 49% of those of the water group, and the root system was short and sparse (average length 1.7 cm). The addition of 12.5 μg/mL TA-Fe nanomaterial increased underground fresh weight (34 mg) and aboveground fresh weight (26 mg) by 21% and 53%, respectively, compared with that of the NaCl group. However, the aboveground length (6.9 cm) was still significantly lower than that of the water group (11.67 cm), indicating that the morphological improvement was limited by the low concentration. Underground fresh weight (36 mg) and aboveground fresh weight (28 mg) of the 50 μg/mL TA-Fe nanomaterial group were increased by 29% and 65%, respectively, compared with that of the NaCl group because the effect was weakened by the decrease in bioavailability of the nanomaterial at the high concentration.

Notably, the best overall performance was observed in the group with the addition of 25 μg/mL TA-Fe nanomaterial, with 43% and 88% enhancement in below-ground fresh weight (40 mg) and above-ground fresh weight (32 mg), respectively, compared with the NaCl group, and above-ground length recovered to 7.8 cm (67% in the water group), highlighting its optimization of salt stress response through enhanced nutrient uptake and partitioning (**Figures 5a, d**).

Regarding dry weight and the ratio of dry-to-fresh weight, the aboveground dry weight of rice seedlings subjected to salinity stress was markedly reduced compared to the other two groups (**Figures 5b, c**). Additionally, the higher dry-to-fresh weight ratio reflects reduced vitality due to salt stress. In contrast, the data for the rice seedlings treated with 25 μg/mL TA-Fe Nanomaterial indicated that it has excellent effects in mitigating the reduction of seedling vitality and inhibiting growth under salt stress.

Additionally, the EB staining results for the aboveground parts of the rice seedlings showed that those in the salt stress group were stained deep blue, indicating substantial damage to the membrane structures of the cells, allowing EB to enter and stain the cells (**Figure 5h**). In contrast, the aboveground parts of the rice seedlings treated with 25 μg/mL TA-Fe Nanomaterial exhibited lighter blue coloration, suggesting less damage from salt stress. The DAB staining outcomes indicated that the aboveground parts of the seedlings in the salt stress group were predominantly stained dark brown, indicating abnormal accumulation of H_2_O_2_ within the cells (**Figure 5i**). In contrast, the seedlings treated with 25 μg/mL TA-Fe Nanomaterial showed mainly light yellow staining, suggesting that this treatment effectively reduced the abnormal accumulation of hydrogen peroxide caused by salt stress, thereby alleviating oxidative damage to the cells.

Salt stress induced pronounced membrane damage and oxidative stress in *Oryza sativa* seedlings, as evidenced by deep blue EB staining (indicative of compromised membrane integrity) and dark brown DAB staining (reflecting H_2_O_2_ overaccumulation) in the NaCl-treated group. Quantitative analyses revealed severely reduced EB-stained areas (41.1–43.5%) and dominant DAB-stained regions (97.1–99.1%), confirming extensive cellular damage. In contrast, supplementation with 25 μg/mL TA-Fe nanomaterials markedly alleviated these effects, yielding lighter EB (light blue) and DAB (light yellow) staining intensities (**Figures 5e, f**). This corresponded to enhanced membrane preservation (EB-stained: 16.2–26.9%) and suppressed H_2_O_2_ levels (DAB-stained: 17.4–31.4%), demonstrating that TA-Fe nanomaterials mitigate salt-induced cytotoxicity by stabilizing membrane structures and scavenging reactive oxygen species, thereby reducing oxidative damage. These findings underscore the efficacy of 25 μg/mL TA-Fe in protecting cellular integrity under salt stress conditions.

The experimental results demonstrated significant variations in membrane integrity and oxidative stress levels among different treatment groups. As shown in **Table 1** (EB staining), the NaCl-stressed group exhibited a markedly elevated OD_610_ value (0.911 ± 0.054), corresponding to a substantial decline in relative cell viability (52.2 ± 2.83%), indicative of severe membrane damage. In contrast, supplementation with 25 μg/mL TA-Fe nanomaterials significantly reduced EB uptake (OD_610_: 0.205 ± 0.004) and restored cell viability to 89.24 ± 0.21%, approaching the water control group (99 ± 0.05%). Similarly, DAB staining revealed a pronounced accumulation of H_2_O_2_ in the NaCl group (OD_450_: 0.089 ± 0.005), while 25 μg/mL TA-Fe treatment effectively attenuated oxidative stress, lowering both OD_450_ (0.053 ± 0.002) and H_2_O_2_ content (59.55 ± 2.25%) to levels comparable with milder stress conditions (**Table 2**).

**Table 1:** Extract solution absorbance from EB stained leaves of *Oryza sativa* and relative cell viability under different groups.

GROUPS	OD_610_	RELATIVE CELL VIABILITY (%)
Water	0.019 ± 0.001	99 ± 0.05
NaCl	0.911 ± 0.054	52.2 ± 2.83
NaCl + 12.5 μ g/mL TA-Fe nanomaterials	0.635 ± 0.031	66.68 ± 1.63
NaCl + 25 μ g/mL TA-Fe nanomaterials	0.205 ± 0.004	89.24 ± 0.21
NaCl + 50 μ g/mL TA-Fe nanomaterials	0.498 ± 0.027	73.87 ± 1.42
Death*	1.906 ± 0.112	0.00

*Heat-killed controls (seedlings treated at 100°C for 15–20 min). Complete methodology in Section 2.6.

**Table 2:** Extract solution absorbance from DAB stained leaves of *Oryza sativa* and relative H_2_O_2_ content under different groups.

GROUPS	OD_450_	RELATIVE H_2_O_2_ CONTENT (%)
Water	0.005 ± 0.001	5.62 ± 1.11
NaCl	0.089 ± 0.005	100
NaCl + 12.5 μ g/mL TA-Fe nanomaterials	0.071 ± 0.004	86.5 ± 2.23
NaCl + 25 μ g/mL TA-Fe nanomaterials	0.053 ± 0.002	59.55 ± 2.25
NaCl + 50 μ g/mL TA-Fe nanomaterials	0.066 ± 0.002	74.16 ± 2.24

In summary, salt stress leads to abnormal accumulation of hydrogen peroxide in the aboveground parts of rice seedlings, compromising the integrity of cell membrane structures and potentially resulting in cell death. Applying 25 μg/mL, TA-Fe Nanomaterial can significantly alleviate oxidative damage to rice seedling cells caused by salt stress, reduce cell death, and thereby ameliorate the suppressive effects of salinity stress on rice seedling growth.

## 4 Discussion

Recent advancements in nanotechnology have highlighted the potential of nanoparticles in mitigating plant stress; however, critical gaps remain in addressing biocompatibility and oxidative risks associated with conventional metal oxide nanomaterials. For instance, while TiO_2_ and ZnO nanoparticles demonstrate ROS scavenging capabilities, their inherent toxicity and environmental persistence limit practical application (Berardis et al., 2010; Rizk et al., 2017). In contrast, our study introduces tannic acid-iron nanomaterial (TA-Fe Nanomaterial) as a novel, biocompatible alternative for alleviating salt stress in rice. By leveraging the synergistic antioxidant properties of tannic acid’s polyphenolic hydroxyl groups and iron’s catalytic activity, TA-Fe Nanomaterial effectively neutralizes ROS—including superoxide anions (·O_2_−), hydroxyl radicals (·OH), and hydrogen peroxide (H_2_O_2_)—while maintaining membrane integrity and nutrient homeostasis (**Figures 2**-**5**). Notably, the removal of ·O_2_− aligns with the natural function of superoxide dismutase (SOD), a key nanomaterial in plant antioxidant defense. Although endogenous SOD activity was not directly measured here, the observed reduction in superoxide anion levels (**Figure 2d**) and alleviation of oxidative damage (**Figure 5h**) suggest a synergistic interaction between the nanomaterial and the plant’s intrinsic SOD system. This is consistent with recent findings by Zhang et al. (2024), who reported that nanomaterials mimicking SOD activity enhance stress tolerance by reducing ROS burden and upregulating endogenous antioxidants.

Our results demonstrate that 25 μ g/mL TA-Fe Nanomaterial enhances rice seed germination by 85% under 100 mM NaCl stress (**Figure 3**), with hydroponic and soil experiments revealing 104% and 69% increases in root length and shoot biomass, respectively (**Figures 4**, **5**). These improvements correlate with reduced H_2_O_2_ accumulation (**Figure 5h**), underscoring the nanomaterial’s ability to mitigate oxidative damage—a critical advancement over CeO_2_ and Fe_2_O_3_ nanoparticles, which often exacerbate oxidative stress at higher concentrations (Sadeghi et al., 2015). Unlike traditional metal oxide nanoparticles that may disrupt SOD-CAT balance, TA-Fe Nanomaterial’s biocompatibility ensures minimal interference with native enzymatic systems, preserving redox homeostasis. This dual efficacy in ROS scavenging and cellular protection addresses the limitations of prior studies focused solely on ROS neutralization.

These findings build upon earlier work by Zhang et al. (2022), who emphasized the importance of ROS-scavenging nanomaterials in plant stress tolerance. However, our study uniquely addresses the dual challenges of efficacy and safety: TA-Fe Nanomaterial’s superior biocompatibility and thermal stability (**Figure 1e**) position it as a sustainable alternative to existing metal oxide nanoparticles. Future research should explore its long-term effects on crop yield and soil microbiota to fully realize its agricultural potential.

## 5 Conclusion

This study demonstrates that 25 μ g/mL TA-Fe Nanomaterial significantly enhances rice growth and antioxidant defense under salt stress. The nanomaterial effectively scavenges ROS, mitigates oxidative damage, and improves plant vitality. Nevertheless, Nevertheless, further research is needed to maintain water and nutrient balance within the crop and to investigate whether TA-Fe Nanomaterial affects the quality and yield of mature crops.

## Data Availability

The original contributions presented in the study are included in the article/supplementary material. Further inquiries can be directed to the corresponding author.

## References

[B1] Aguilera J. R. Venegas V. Oliva J. M. Sayagués M. J. Miguel M. Sánchez-Alcázar J. A.. (2016). Targeted multifunctional tannic acid nanoparticles. RSC Adv. 6, 7279-7287. doi: 10.1039/C5RA19405A

[B2] Akanbi-Gada M. A. Ogunkunle C. O. Vishwakarma V. Viswanathan K. Fatoba P. O. (2019). Phytotoxicity of nano-zinc oxide to tomato plant (Solanum lycopersicum L.): Zn uptake, stress enzymes response and influence on non-enzymatic antioxidants in fruits. “Environmental Technol. Innovation 14, 100325-100325. doi: 10.1016/j.eti.2019.100325

[B3] Ali B. Saleem M. H. Ali S. Shahid M. Sagir M. Tahir M. B.. (2022). Mitigation of salinity stress in barley genotypes with variable salt tolerance by application of zinc oxide nanoparticles. Front. Plant Sci. 13. doi: 10.3389/fpls.2022.973782, PMID: 36072329 PMC9441957

[B4] Baker C. J. Mock N. M. (1994). An improved method for monitoring cell death in cell suspension and leaf disc assays using evans blue. Plant Cell Tissue Organ Culture 39, 7–12. doi: 10.1007/BF00037585

[B5] Berardis B. D. Li Y. Zhang H. Qin P. Hu X. Wang J.. (2010). Exposure to ZnO nanoparticles induces oxidative stress and cytotoxicity in human colon carcinoma cells. “Toxicology Appl. Pharmacol. 246 (3), 116–127. doi: 10.1016/j.taap.2010.04.012, PMID: 20434478

[B6] Chen M. Mock N. M. (2021). Combined organic amendments and mineral fertilizer application increase rice yield by improving soil structure, P availability and root growth in saline-alkaline soil. Soil Tillage Res. 212. doi: 10.1016/j.still.2021.105060

[B7] Cheng S. Cao L. Zhuang J. Chen S. Zhan X. Fan Y.. (2007). Super Hybrid Rice Breeding in China: Achievements and Prospect. Journal of Integrative Plant Biology 49 (6), 805–810. doi: 10.1111/j.1744-7909.2007.00514.x

[B8] Cui Z. Zhang S. Liu L. Wu L. Ding X. (2022). Lighting up agricultural sustainability in the new era through nanozymology: an overview of classifications and their agricultural applications. J. Agric. Food Chem. vol 70, 13445–13463. doi: 10.1021/acs.jafc.2c04882, PMID: 36226740

[B9] Dondurmacıoğlu F. Avan A. N. Apak R. (2017). Simultaneous detection of superoxide anion radicals and determination of the superoxide scavenging activity of antioxidants using a N,N-dimethyl-p-phenylene diamine/Nafion colorimetric sensor. Analytical Methods 9 (6), 6202-6212. doi: 10.1039/C7AY02132A

[B10] Forney L. J. Reddy C. A. Pankratz H. S. (1982). Ultrastructural localization of hydrogen peroxide production in ligninolytic phanerochaete chrysosporium cells. Appl. Environ. Microbiol. 44, 732–736. doi: 10.1128/aem.44.3.732-736.1982, PMID: 16346099 PMC242083

[B11] Gupta A. Fareha R. Richa M. Manikant T. Neelam P. (2023). Nanotechnology applications in sustainable agriculture: An emerging eco-friendly approach. “Plant Nano Biol. 4, 100033. doi: 10.1016/j.plana.2023.100033

[B12] Hamza T. A. Hadwan M. H. (2020). New spectrophotometric method for the assessment of catalase enzyme activity in biological tissues. “Current Analytical Chem. 16, 1054–1062. doi: 10.2174/1573411016666200116091238

[B13] Hou D. Cui X. Liu M. Qie H. Tang Y. Xu R.. (2024). The effects of iron-based nanomaterials (Fe NMs) on plants under stressful environments: Machine learning-assisted meta-analysis. J. Environ. Manage. 354, 120406. doi: 10.1016/j.jenvman.2024.120406, PMID: 38373376

[B14] Ismail A. M. Horie T. (2017). Genomics, physiology, and molecular breeding approaches for improving salt tolerance. Annu. Rev. Plant Biol. 68, 405–434. doi: 10.1146/annurev-arplant-042916-040936, PMID: 28226230

[B15] Janicka M. Chodkowski M. Osinska A. Bylinska K. Obuch-Woszczatyńska O. Patrycy M.. (2025). Adjuvanticity of Tannic Acid-Modified Nanoparticles Improves Effectiveness of the Antiviral Response. Int J Nanomedicine 20, 3977–3977. doi: 10.2147/ijn.s512509, PMID: 40191045 PMC11972000

[B16] Kaczmarek-Szczepańska B. Zasada L. Michalska-Sionkowska M. Vishnu J. Manivasagam G. (2023). The modification of titanium surface by decomposition of tannic acid coating. “Applied Sci. 13, 8. doi: 10.3390/app13085204

[B17] Kourgialas N. N. Karatzas G. P. Koubouris G. C. (2017). A GIS policy approach for assessing the effect of fertilizers on the quality of drinking and irrigation water and wellhead protection zones (Crete, Greece). J. Environ. Manage. 189, 150–159. doi: 10.1016/j.jenvman.2016.12.038, PMID: 28013089

[B18] Letelier ME. Molina-Berríos A. Cortés-Troncoso J. Jara-Sandoval J. Holst M. Palma K.. (2008). DPPH and oxygen free radicals as pro-oxidant of biomolecules. Toxicol. vitro: an Int. J. published Assoc. BIBRA 22, 279–286. doi: 10.1016/j.tiv.2007.08.002, PMID: 17888621

[B19] Liu M. Pan T. Allakhverdiev S. I. Yu M. Shabala S. (2020). Crop halophytism: an environmentally sustainable solution for global food security. Trends Plant Sci. 25, 630–634. doi: 10.1016/j.tplants.2020.04.008, PMID: 32444156

[B20] Liu Y. Xiao Z. Chen F. Yue L. Zou H. Lyu J.. (2021). Metallic oxide nanomaterials act as antioxidant nanozymes in higher plants: Trends, meta-analysis, and prospect. Sci. total Environ. 780, 146578. doi: 10.1016/j.scitotenv.2021.146578, PMID: 34030327

[B21] Liu Y. Cao X. Yue L. Wang C. Tao M. Wang Z.. (2022). Foliar-applied cerium oxide nanomaterials improve maize yield under salinity stress: Reactive oxygen species homeostasis and rhizobacteria regulation. Environ. pollut. (Barking Essex: 1987) 299, 118900. doi: 10.1016/j.envpol.2022.118900, PMID: 35085650

[B22] Marić I. Zore A. Rojko F. Škapin A. S. Štukelj R. Učakar A. (2023). Antifungal Effect of Polymethyl Methacrylate Resin Base with Embedded Au Nanoparticles. Nanomaterials (Basel) 13 (14), 2128. doi: 10.3390/nano13142128, PMID: 37513139 PMC10383817

[B23] Markus K. Kirschbaum T. Metzsch-Zilligen E. Pfaendner R. (2025). Processing stability and radical scavenging efficiency of novel biobased stabilizers: Insights from long-term extrusion and DPPH assays. Polymer Degradation Stability 233. doi: 10.1016/j.polymdegradstab.2024.111162

[B24] Maswada H. F. Djanaguiraman M. Prasad P. V. V. (2018). Seed treatment with nano-iron (III) oxide enhances germination, seeding growth and salinity tolerance of sorghum. “Journal Agron. Crop Sci. 204, 6. doi: 10.1111/jac.12280

[B25] Miller G. Suzuki N. Ciftci-Yilmaz S. Mittler R. (2010). Reactive oxygen species homeostasis and signalling during drought and salinity stresses. Plant Cell Environ. 33, 453–467. doi: 10.1111/j.1365-3040.2009.02041.x, PMID: 19712065

[B26] Mittler R. Zandalinas S. I. Fichman Y. Breusegem F. V. (2022). Reactive oxygen species signalling in plant stress responses. Nat. Rev. Mol. Cell Biol. 23, 663–679. doi: 10.1038/s41580-022-00499-2, PMID: 35760900

[B27] Mustafa N. Raja N. I. Ilyas N. Abasi F. Ahmad M. S. Ehsan M.. (2022). Exogenous application of green titanium dioxide nanoparticles (TiO2 NPs) to improve the germination, physiochemical, and yield parameters of wheat plants under salinity stress. Molecules (Basel Switzerland) 27, 4884. doi: 10.3390/molecules27154884, PMID: 35956833 PMC9370171

[B28] Pucci C. Martinelli C. De Pasquale D. Battaglini M. Leo N. Degl'Innocenti A.. (2022). Tannic Acid-Iron Complex-Based Nanoparticles as a Novel Tool against Oxidative Stress. ACS Appl Mater InterfacesACS Appl Mater Interfaces 14 (14), 15927–15941. doi: 10.1021/acsami.1c24576, PMID: 35352893 PMC9011352

[B29] Rawat N. Wungrampha S. Singla-Pareek S. L. Yu M. Shabala S. Pareek A. (2022). Rewilding staple crops for the lost halophytism: Toward sustainability and profitability of agricultural production systems. Mol. Plant 15, 45–64. doi: 10.1016/j.molp.2021.12.003, PMID: 34915209

[B30] Revathy R. Sajini T. Augustine C. Joseph N. (2023). Iron-based magnetic nanomaterials: Sustainable approaches of synthesis and applications. Results Eng. 18, 101114. doi: 10.1016/j.rineng.2023.101114

[B31] Rizk M. Z. Ali S. A. Hamed M. A. El-Rigal N. S. Aly H. F. Salah H. H. (2017). Toxicity of titanium dioxide nanoparticles: Effect of dose and time on biochemical disturbance, oxidative stress and genotoxicity in mice. Biomedicine Pharmacotherapy 90, 466-472. doi: 10.1016/j.biopha.2017.03.089, PMID: 28391168

[B32] Rossi L. Zhang W. Lombardini L. Ma X. (2016). The impact of cerium oxide nanoparticles on the salt stress responses of Brassica napus L. Environ. pollut. (Barking Essex: 1987) 219, 28–36. doi: 10.1016/j.envpol.2016.09.060, PMID: 27661725

[B33] Rossi L. Zhang W. Ma X. (2017). Cerium oxide nanoparticles alter the salt stress tolerance of Brassica napus L. by modifying the formation of root apoplastic barriers. Environ. pollut. (Barking Essex: 1987) 229, 132–138. doi: 10.1016/j.envpol.2017.05.083, PMID: 28582676

[B34] Rutely C. B. C. Jean-M F. Walter Z. T. Xochitl D. B. Mika S. (2018). Towards reliable quantification of hydroxyl radicals in the Fenton reaction using chemical probes. RSC Adv. 8, 5321–5330. doi: 10.1039/c7ra13209c, PMID: 35542446 PMC9078104

[B35] Sadeghi L. Tanwir F. Babadi V. Y. (2015). *In vitro* toxicity of iron oxide nanoparticle: Oxidative damages on Hep G2 cells. Exp. Toxicologic Pathol. 67 (2), 197-203. doi: 10.1016/j.etp.2014.11.010, PMID: 25497787

[B36] Sahab S. Suhani I. Srivastava V. Chauhan P. S. Singh R. P. Prasad V. (2021). Potential risk assessment of soil salinity to agroecosystem sustainability: Current status and management strategies. Sci. total Environ. 764, 144164. doi: 10.1016/j.scitotenv.2020.144164, PMID: 33385648

[B37] Sahiner N. Sagbas S. Aktas N. Silan C. (2016). Inherently antioxidant and antimicrobial tannic acid release from poly(tannic acid) nanoparticles with controllable degradability.” Colloids and surfaces. B Biointerfaces 142, 334–343. doi: 10.1016/j.colsurfb.2016.03.006, PMID: 26970821

[B38] Sahiner N. Sengel S. B. (2016). Tannic acid decorated poly(methacrylic acid) micro and nanoparticles with controllable tannic acid release and antioxidant properties. Colloids Surfaces A: Physicochemical Eng. Aspects 508, 30-38. doi: 10.1016/j.colsurfa.2016.08.014

[B39] Song Y. Zheng C. Li S. Chen J. Jiang M. (2023). Chitosan-magnesium oxide nanoparticles improve salinity tolerance in rice (*Oryza sativa* L.). ACS Appl. materials interfaces 15, 20649–20660. doi: 10.1021/acsami.3c00043, PMID: 37078774

[B40] Souza L. R. R. Bernardes L. E. Barbetta M. F. S. Veiga M. A. M. S. (2019). Iron oxide nanoparticle phytotoxicity to the aquatic plant Lemna minor: effect on reactive oxygen species (ROS) production and chlorophyll a/chlorophyll b ratio. Environ. Sci. pollut. Res. Int. 26, 24121–24131. doi: 10.1007/s11356-019-05713-x, PMID: 31228067

[B41] Sui X. Wang J. Zhao Z. Liu B. Liu M. Liu M.. (2024). Phenolic compounds induce ferroptosis-like death by promoting hydroxyl radical generation in the Fenton reaction. Commun. Biol. 7, 199. doi: 10.1038/s42003-024-05903-5, PMID: 38368473 PMC10874397

[B42] Teige M. Scheikl E. Eulgem T. Dóczi R. Ichimura K. Shinozaki K.. (2004). The MKK2 pathway mediates cold and salt stress signaling in Arabidopsis. Mol. Cell 15, 141–152. doi: 10.1016/j.molcel.2004.06.023, PMID: 15225555

[B43] Van Noorden C. J. Butcher R. G. (1989). Development of the "Third-Generation" Hybrid Rice in China. Analytical Biochem. 176, 170–174. doi: 10.1016/0003-2697(89)90288-1, PMID: 2540673

[B44] Wang H. Deng X. W. (2018). Development of the "Third-Generation" Hybrid Rice in China. Genomics Proteomics Bioinformatics 16 (6), 393–396. doi: 10.1016/j.gpb.2018.12.001, PMID: 30553883 PMC6411946

[B45] Wungrampha S. Joshi R. Singla-Pareek S. L. Pareek A. (2018). Photosynthesis and salinity: are these mutually exclusive? Photosynthetica 56, 366–381. doi: 10.1007/s11099-017-0763-7

[B46] Yan W. Shi M. Dong C. Liu L. Gao C. (2020). Applications of tannic acid in membrane technologies: A review. Adv Colloid Interface Sci. 284, 102267. doi: 10.1016/j.cis.2020.102267, PMID: 32966965

[B47] Yang Z. Wang C. Xue Y. Liu X. Chen S. Song C. P.. (2019). Calcium-activated 14-3–3 proteins as a molecular switch in salt stress tolerance. Nat. Commun. 10, 1199. doi: 10.1038/s41467-019-09181-2, PMID: 30867421 PMC6416337

[B48] Yang Y. Han X. Ma L. Wu Y. Liu X. Fu H.. (2021). Dynamic changes of phosphatidylinositol and phosphatidylinositol 4-phosphate levels modulate H+-ATPase and Na+/H+ antiporter activities to maintain ion homeostasis in Arabidopsis under salt stress. Mol. Plant 14, 2000–2014. doi: 10.1016/j.molp.2021.07.020, PMID: 34339895

[B49] Zhang Y. Fu L. Jeon S. J. Yan J. Giraldo J. P. Matyjaszewski K.. (2022). Star polymers with designed reactive oxygen species scavenging and agent delivery functionality promote plant stress tolerance. ACS nano 16, 4467–4478. doi: 10.1021/acsnano.1c10828, PMID: 35179875

[B50] Zhang C. Shi S. Feng J. Wang T. Liang Y. Du T.. (2024). Cu (II)-chelated ovalbumin mimicking the active centre of superoxide dismutase: Structure, antioxidant and antibacterial properties for food preservation application. Int J Biol. Macromol. 277 (Pt 1), 134090. doi: 10.1016/j.ijbiomac.2024.134090, PMID: 39053832

[B51] Zhao L. Bai T. Wei H. Gardea-Torresdey J. L. Keller A. White J. C. (2022). Nanobiotechnology-based strategies for enhanced crop stress resilience. Nat. Food 3, 829–836. doi: 10.1038/s43016-022-00596-7, PMID: 37117882

